# Predictors of postoperative hypocalcemia occurring after a total thyroidectomy: results of prospective multicenter study

**DOI:** 10.1186/s12893-018-0387-2

**Published:** 2018-08-09

**Authors:** Vitalijus Eismontas, Algirdas Slepavicius, Vinsas Janusonis, Paulius Zeromskas, Virgilijus Beisa, Kestutis Strupas, Zilvinas Dambrauskas, Antanas Gulbinas, Arvydas Martinkenas

**Affiliations:** 1 Department of Abdominal and Endocrine Surgery, Klaipeda University Hospital, Liepojos St. 41, 92288 Klaipeda, Lithuania; 2 Centre of Abdominal Surgery, Vilnius University Hospital Santaros Klinikos, Vilnius, Lithuania; 3 Department of Surgery, Hospital of Lithuanian University of Health Sciences, Kaunas, Lithuania; 4 Department of Medical Technologies, Faculty of Health Sciences, Klaipeda University, Klaipeda, Lithuania

**Keywords:** Total thyroidectomy, Hypocalcemia, Thyroid surgery, Predictors

## Abstract

**Background:**

Thyroid surgeries are among the most common operations performed in the world. Hypocalcemia following total thyroidectomy is a common complication that is sometimes difficult to correct. The aim of this study is to determine the risk factors for hypocalcemia following total thyroidectomy and their clinical value.

**Methods:**

From January 2015 through to April 2017, 400 patients were included in this prospective multicenter study. All patients underwent total thyroidectomy due to various thyroid diseases. The following risk factors were analyzed: pre-operative and post-operative biochemical blood parameters, clinical effects and factors related to surgery, the patient, and the disease.

**Results:**

Post-operative hypocalcemia developed in 257 patients (64.2%). Of them, 197 patients (76.7%) were diagnosed with asymptomatic hypocalcemia. Clinical symptoms were present in 60 of the 257 patients with hypocalcemia (23.3%). The statistically significant predictors of hypocalcemia were decreased calcium and ionized calcium pre-operatively (*p* < 0.001), parathyroid hormone on day one following surgery (*p* < 0.001), thyrotoxicosis <10 years before surgery (odds ratio 1.65, 95% CI 1.01–2.70, *p* = 0.046), the number of parathyroid glands found during surgery (odds ratio 0.52, 95% CI 0.38–0.70, *p* < 0.001), ligation of the trunk of the left inferior thyroid artery (odds ratio 2.04, 95% CI 1.27–3.29, *p* = 0.003), ligation of the trunk of the right inferior thyroid artery (odds ratio 2.37, 95% CI 1.47–3.81, *p* < 0.001), and the number of transplanted parathyroid glands (odds ratio 1.87, 95% CI 1.12–2.97, *p* = 0.015). In the multivariate analysis, age (odds ratio 1.05, 95% CI 1.01–1.09, *p* = 0.029) and gender (odds ratio 5.94, 95% CI 1.13–31.26, *p* = 0.035) were statistically significant predictors.

**Conclusions:**

This study demonstrates that there is a number of different patient (gender, age, and duration of thyrotoxicosis <10 years before surgery) and surgical (number of parathyroid glands found during surgery, decreased calcium and ionized calcium before surgery, parathyroid hormone on day one following surgery, and ligation of the trunk of the left and right inferior thyroid artery) risk factors predictive of hypocalcemia following total thyroidectomy. Optimization of the surgical technique could possibly prevent the occurrence of hypocalcemia after total thyroidectomy in some cases; in other cases, identification of known risk factors post-operatively could permit early detection and effective treatment of these patients.

## Background

Thyroid surgeries are among the most common in the world [1]. Thyroid surgery is the definitive management option for thyroid malignancies, and also for benign diseases such as multinodular goiter with compression symptoms [2]. Hypocalcemia following total thyroidectomy (TT) is a relatively frequent complication, which is sometimes difficult to correct. Temporary hypocalcemia occurs in 50–68% of post-TT patients [3, 4], while permanent hypocalcemia occurs in 3% of post-TT patients [5–7]. Temporary hypocalcemia is defined by various authors as a post-surgery decrease in calcium (Ca), lasting for 6 to 12 months; permanent hypocalcemia is a post-TT decrease in Ca lasting for more than 12 months [8]. Post-TT hypocalcemia depends on a number of factors, including biochemical blood parameters before and after surgery, clinical effects and factors related to surgery, surgical technique, surgeon’s experience, the patient, and the disease [9].

The aim of this study was to determine the risk factors for postoperative hypocalcemia following TT and their clinical value.

## Methods

From January 2015 to April 2017, 400 patients underwent surgeries for various thyroid diseases at Klaipėda University Hospital, Lithuania and Vilnius University Hospital Santaros Klinikos, Lithuania. Permission from the Lithuanian Bioethics Committee was obtained for this prospective multicenter research (2014-12-02, No.: L-14-09/1, No.: L-14-09/2). All patients signed a consent form before surgery. The inclusion criteria for this study were thyroid nodules in 361 patients (90.3%), thyroid carcinoma in 84 patients (21%), thyroiditis in 37 patients (9.3%). Some patients had more than one indication for surgery. The exclusion criteria for this study were previous hemithyroidectomy, lobectomy, resection of thyroid gland, and diagnosed pathology of parathyroid glands (PGs). In this study all operations were performed by a three high-volume thyroid surgeons. Various factors that may influence post-TT hypocalcemia were investigated, including pre-operative and post-operative biochemical blood parameters, clinical effects and factors related to surgery, the patient, and the disease. Postoperative hypocalcemia was diagnosed when the level of Ca in the blood was <2.10 mmol/L. Before surgery, Ca, ionized calcium (iCa), parathyroid hormone (PTH), 25-hydroxyvitamin D (25-hydroxy Vit D), and thyroid hormones were measured. Length of stay in hospital for patients following the surgery was 2 days. Measurements following TT on the first and second day at six oʼclock in the morning were performed. On day one following the surgery, Ca, iCa, and PTH were measured. On day two following the surgery, Ca, iCa were measured. Clinical expression of post-operative hypocalcemia, and the time when the disease manifested, were assessed. The impact of surgery-related factors on post-operative hypocalcemia was analyzed, including the number of PGs observed during the surgery, number of autotransplanted PGs, and ligation of vessels supplying blood to the thyroid gland. Oral Ca with/without calcitriol were not routinely given unless patients had symptoms or serum Ca < 2 mmol/L. Oral Ca 1500–3000 mg with/without calcitriol 0.25 mcg twice daily were appointed for 2 weeks following the surgery. Calcitriol should be given in case a higher risk of postoperative hypocalcemia, including recurrent neck surgery, central neck dissection, intraoperative PG injury, autotransplanted PGs, also for patients with low postoperative PTH and 25-hydroxy Vit D deficiency.

All blood samples were taken from a peripheral vein. 25-hydroxy Vit D and PTH were measured with Cobas e411 analyzer (Roche Diagnostics, Mannheim, Germany). The normal range of 25-hydroxy Vit D in the blood is 75.00–106.75 nmol/L for men and 75.00–124.75 nmol/L for women. The normal PTH range is 15.00–65.00 pg/mL. The level of Ca and iCa was determined with Architect ci8200 analyzer (Abbot, Wiesbaden, Germany).

The normal range of Ca in the blood is 2.10–2.55 mmol/L and the normal range of iCa in the blood is 1.00–1.30 mmol/L.

### Statistical analysis

Data analysis was performed using SPSS version 24.0 and MS Excel 2016. Univariate descriptive statistics were performed for the continuous data. Categorical data were summarized as frequencies and percentages. The chi-square test or the Fishers exact test was used to estimate the associations between categorical variables. Comparison of proportions between the groups was performed using the z test. After testing for normality, parametric or non-parametric tests were performed, as required. Comparison of health variables between hypocalcemia (no (0), yes (1)) groups was performed using the Students *t* test and the Mann–Whitney test. ANOVA and the Kruskal–Wallis test was employed for comparison of two or more groups. Univariate binary logistic regression analysis was used to determine the relative significance of sociodemographic and perceived health variables as predictors of hypocalcemia. Variables associated with post-operative hypocalcemia in univariate analyses were incorporated into a multivariate logistic regression to identify independent risk factors for post-operative hypocalcemia following thyroidectomy. Multivariate logistic regression analysis, using the backward LR (Likelihood Ratio) test, was employed to remove variables with *p* > 0.1. Odds ratios (ORs) of statistically significant predictors are presented with 95% confidence intervals (CIs). A *p* value of less than 0.05 was taken to indicate statistical significance.

## Results

From January 2015 to April 2017, 400 patients underwent TT’s. There were 349 women (87.2%) and 51 men (12.8%). The mean age was 57.2 years and the mean weight was 79.2 kg. According to the data, post-operative hypocalcemia developed in 257 patients (64.2%). Of them, 197 patients (76.7%) were diagnosed with asymptomatic hypocalcemia. Clinical symptoms were observed in 60 patients (23.3%) of the 257 patients with hypocalcemia. Numbness of fingers was observed in 58 patients (22.6% of the patients with post-operative hypocalcemia). Chvostek’s signs were present in 14 patients (5.4% of the patients with post-operative hypocalcemia). Trousseau’s signs were present in eight patients (3.1% of the patients with post-operative hypocalcemia). In the group of patients with post-operative hypocalcemia, one symptom was present in 44 patients (17.1%), two symptoms were present in 11 patients (4.3%), and three symptoms were observed in five patients (1.9%). See Tables 1 and 2 for details.

**Table 1:** Patient demographics and clinical characteristics

Variable^a^	Value
Gender, *n* (%)	400 (100)
Female	349 (87.2)
Male	51 (12.8)
Age in years, mean (SD)	57.2 (13.4)
Weight in kg, mean (SD)	79.2 (15.8)
Thyrotoxicosis before surgery, *n* (%)	
No	277 (69.2)
<10 years	105 (26.3)
≥10 years	18 (4.5)
Graves’ disease, *n* (%)	
No	379 (94.7)
Yes	21 (5.3)
Recurrent goiter, *n* (%)	
No	397 (99.2)
Yes	3 (0.8)
Retrosternal goiter, *n* (%)	
No	393 (98.2)
Yes	7 (1.8)
Bone mass density, T level, *n* (%)	
No	394 (98.5)
Yes	6 (1.5)
Preoperative calcium, mmol/L, mean (SD)	2.36 (0.10)
MD (IQR)	2.36 (0.12)
Preoperative ionized calcium, mmol/L, mean (SD)	1.06 (0.08)
MD (IQR)	1.04 (0.07)
Preoperative PTH, ng/L (pg/mL), mean (SD)	57.71 (22.27)
MD (IQR)	53.90 (26.63)
Preoperative 25-hydroxyvitamin D, nmol/L, mean (SD)	45.27 (22.03)
MD (IQR)	41.78 (29.72)
Preoperative TSH, mlU/L, mean (SD)	0.94 (1.22)
MD (IQR)	0.75 (1.22)
Preoperative FT3, pmol/L, mean (SD)	4.87 (2.36)
MD (IQR)	4.66 (0.84)
Preoperative FT4, pmol/L, mean (SD)	13.31 (2.69)
MD (IQR)	13.43 (2.71)
Thyroidectomy without lymphadenectomy, *n* (%)	400 (100)
No	66 (16.5)
Yes	334 (83.5)
Thyroidectomy with one sided central lymphadenectomy, *n* (%)	
No	344 (86.0)
Yes	56 (14.0)
Thyroidectomy with central lymphadenectomy on both sides, *n* (%)	
No	390 (97.5)
Yes	10 (2.5)
Number of observed parathyroid glands, *n* (%)	
0	2 (0.5)
1	8 (2.0)
2	97 (24.2)
3	208 (52.0)
4	85 (21.3)
Upper branch of left inferior thyroid artery, *n* (%)	
Non-ligation	363 (90.7)
Ligation	37 (9.3)
Lower branch of left inferior thyroid artery, *n* (%)	
Non-ligation	366 (91.5)
Ligation	34 (8.5)
Trunk of left inferior thyroid artery, *n* (%)	
Non-ligation	90 (22.5)
Ligation	310 (77.5)
Upper branch of right inferior thyroid artery, *n* (%)	
Non-ligation	358 (89.5)
Ligation	42 (10.5)
Lower branch of right inferior thyroid artery, *n* (%)	
Non-ligation	366 (91.5)
Ligation	34 (8.5)
Trunk of right inferior thyroid artery, *n* (%)	
Non-ligation	91 (22.8)
Ligation	309 (77.2)
Number of darkened parathyroid glands at the end of surgery, *n* (%)	
0	305 (76.2)
1	80 (20.0)
2	9 (2.3)
3	6 (1.5)
Parathyroid gland hematoma, *n* (%)	
No	379 (94.7)
Yes	21 (5.3)
Number of autotransplanted parathyroid glands, *n* (%)	
0	332 (83.0)
1	54 (13.4)
2	11 (2.8)
3	3 (0.8)
Adenomatous nodules, *n* (%)	
No	334 (83.5)
Yes	66 (16.5)
Colloidal nodules, *n* (%)	
No	130 (32.5)
Yes	270 (67.5)
Papillary carcinoma, *n* (%)	
No	320 (80.0)
Yes	80 (20.0)
Follicular carcinoma, *n* (%)	
No	399 (99.7)
Yes	1 (0.3)
Medullary carcinoma, *n* (%)	
No	397 (99.3)
Yes	3 (0.8)
Anaplastic carcinoma, *n* (%)	
No	400 (100)
Autoimmune thyroiditis, *n* (%)	
No	363 (90.7)
Yes	37 (9.3)
Calcium day one, mmol/L, mean (SD)	2.12 (0.14)
MD (IQR)	2.11 (0.20)
Ionized calcium day one, mmol/L, mean (SD)	0.99 (0.08)
MD (IQR)	0.99 (0.10)
PTH day one, ng/L (pg/mL), mean (SD)	29.98 (19.46)
MD (IQR)	29.06 (27.61)
Calcium day two, mmol/L, mean (SD)	2.05 (0.16)
MD (IQR)	2.06 (0.22)
Ionized calcium day two, mmol/L, mean (SD)	0.98 (0.09)
MD (IQR)	0.98 (0.11)
Revision for bleeding, *n* (%)	
No	396 (99.0)
Yes	4 (1.0)
Wound infection, *n* (%)	
No	400 (100)
Wound hematoma, *n* (%)	
No	389 (97.2)
Yes	11 (2.8)
Bleeding during surgery, *n* (%)	
No	399 (99.7)
Yes	1 (0.3)
Clinical voice hoarseness, *n* (%)	
No	384 (96.0)
Yes	16 (4.0)
Recurrent laryngeal nerve paralysis on one side, ORL, *n* (%)	
No	392 (98.0)
Yes	8 (2.0)
Recurrent laryngeal nerve paralysis on both sides, ORL, *n* (%)	
No	400 (100)
Clinical manifestation of hypocalcemia, *n* (%)	
None	340 (85.0)
Day one	14 (3.5)
Day two	46 (11.5)
Hypocalcemia without clinical manifestation, *n* (%)	
No	203 (50.7)
Yes	197 (49.3)
Hypocalcemia - Chvostekʼs symptoms, *n* (%)	
No	386 (96.5)
Yes	14 (3.5)
Hypocalcemia - Trousseau’s symptoms, *n* (%)	
No	392 (98.0)
Yes	8 (2.0)
Hypocalcemia - Numbness of fingers, *n* (%)	
No	342 (85.5)
Yes	58 (14.5)
Hypocalcemia, *n* (%)	
No	143 (35.8)
Yes	257 (64.2)
Treatment commenced in hospital with calcium and vitamin D preparations after surgery, *n* (%)	
None	161 (40.3)
Day 1	15 (3.8)
Day 2	33 (8.3)
Day 3	5 (1.3)
On discharge	186 (46.5)

^a^Continuous variables are presented as means and standard deviations (*SD*) & medians (*MD*) and interquartile ranges (*IQR*); categorical variables are presented as *n* (%). *PTH* Parathyroid hormone, *TSH* thyroid stimulating hormone; *FT3* free triiodothyronine, *FT4* free thyroxine, *ORL* otorhinolaryngologist

**Table 2:** Patient characteristics

Variables^a^	Hypocalcemia		*p* value
	Yes	No	
Gender, *n* (%)	257	143	
Female	221 (63.3)	128 (36.7)	*χ2* = 1.02, df = 1, *p* = 0.351
Male	36 (70.6)	15 (29.4)	
Age in years, mean (SD)	57.8 (13.2)	56.0 (13.8)	0.207
Weight, kg., mean (SD)	79.0 (15.2)	79.5 (16.8)	0.721
Thyrotoxicosis before surgery, *n* (%)			
No	170 (66.1)	107 (74.8)	*χ2* = 4.10, df = 2, *p* = 0.129
<10 years	76 (29.6)	29 (20.3)	
≥10 years	11 (4.3)	7 (4.9)	
Graves’ disease, *n* (%)			
No	241 (93.8)	138 (96.5)	*χ2* = 1.38, df = 1, *p* = 0.350
Yes	16 (6.2)	5 (3.5)	
Recurrent goiter, *n* (%)			
No	254 (98.8)	143 (100)	*χ2* = 1.68, df = 1, *p* = 0.556
Yes	3 (1.2)	0	
Retrosternal goiter, *n* (%)			
No	250 (97.3)	143 (100)	*χ2* = 3.96, df = 1, *p* = 0.053
Yes	7 (2.7)	0	
Bone mass density, T level, *n* (%)			
No	394 (98.5)	394 (98.5)	*χ2* = 0.54, df = 1, *p* = 0.671
Yes	6 (1.5)	6 (1.5)	
Preoperative calcium, mmol/L, mean (SD)^b^	2.35 (0.09)	2.39 (0.11)	< 0.001
MD (IQR)	2.35 (0.11)	2.39 (0.12)	
Preoperative ionized calcium, mmol/L, mean (SD)^b^	1.04 (0.07)	1.08 (0.10)	< 0.001
MD (IQR)	1.03 (0.07)	1.07 (0.09)	
Preoperative PTH, ng/L (pg/mL), mean (SD)^b^	57.86 (22.18)	57.43 (22.50)	0.855
MD (IQR)	54.17 (26.38)	53.65 (26.05)	
Preoperative 25-hydroxyvitamin D, nmol/L, mean (SD)^c^	44.22 (22.97)	47.16 (20.17)	0.053
MD (IQR)	40.35 (30.47)	46.40 (26.69)	
Preoperative TSH, mlU/L, mean (SD)^c^	1.12 (1.33)	0.96 (0.97)	0.355
MD (IQR)	0.80 (1.20)	0.70 (1.17)	
Preoperative FT3, pmol/L, mean (SD)^b^	4.85 (2.73)	4.90 (1.50)	0.854
MD (IQR)	4.60 (0.83)	4.71 (0.93)	
Preoperative FT4, pmol/L, mean (SD)^b^	13.53 (2.47)	13.67 (3.05)	0.626
MD (IQR)	13.45 (2.92)	13.40 (2.36)	
Thyroidectomy without lymphadenectomy, *n* (%)			
No	48 (18.7)	18 (12.6)	*χ2* = 2.47, df = 1, *p* = 0.124
Yes	209 (81.3)	125 (87.4)	
Thyroidectomy with one sided central lymphadenectomy, *n* (%)			
No	216 (84.0)	128 (89.5)	*χ2* = 2.28, df = 1, *p* = 0.175
Yes	41 (16.0)	15 (10.5)	
Thyroidectomy with central lymphadenectomy on both sides, *n* (%)			
No	250 (97.3)	140 (97.9)	*χ2* = 0.15, df = 1, *p* = 1.000
Yes	7 (2.7)	3 (2.1)	
Number of observed parathyroid glands, *n* (%)			
0	2 (0.8)	0	*χ2* = 35.23, df = 4, *p* < 0.001
1	3 (1.2)	5 (3.5)	
2	76 (29.6)^**^	21 (14.7)^**^	
3	142 (55.3)	66 (46.2)	
4	34 (13.2)^***^	51 (36.7)^***^	
Upper branch of left inferior thyroid artery, *n* (%)			
Non-ligation	231 (89.9)	132 (92.3)	*χ2* = 0.64, df = 1, *p* = 0.475
Ligation	26 (10.1)	11 (7.7)	
Lower branch of left inferior thyroid artery, *n* (%)			
Non-ligation	232 (90.3)	134 (93.7)	*χ2* = 1.39, df = 1, *p* = 0.267
Ligation	25 (9.7)	9 (6.3)	
Trunk of left inferior thyroid artery, *n* (%)			
Non-ligation	46 (17.9)	44 (30.8)	*χ2* = 8.73, df = 1, *p* = 0.003
Ligation	211 (82.1)	99 (69.2)	
Upper branch of right inferior thyroid artery, *n* (%)			
Non-ligation	232 (90.3)	126 (88.1)	*χ2* = 0.46, df = 1, *p* = 0.501
Ligation	25 (9.7)	17 (11.9)	
Lower branch of right inferior thyroid artery, *n* (%)			
Non-ligation	231 (89.9)	135 (94.4)	*χ2* = 2.42, df = 1, *p* = 0.137
Ligation	26 (10.1)	8 (5.6)	
Trunk of right inferior thyroid artery, *n* (%)			
Non-ligation	44 (17.1)	47 (32.9)	*χ2* = 12.96, df = 1, *p* < 0.001
Ligation	213 (82.9)	96 (67.1)	
Number of darkened parathyroid glands at the end of surgery, *n* (%)			
0	194 (75.5)	111 (77.6)	*χ2* = 3.64, df = 3, *p* = 0.303
1	54 (21.0)	26 (18.2)	
2	7 (2.7)	2 (1.4)	
3	2 (0.8)	4 (2.8)	
Parathyroid gland hematoma, *n* (%)			
No	244 (94.9)	135 (94.4)	*χ2* = 0.05, df = 1, *p* = 0.818
Yes	13 (5.1)	8 (5.6)	
Number of autotransplanted parathyroid glands, *n* (%)			
0	205 (79.8)^*^	127 (88.8)^*^	*χ2* = 6.32, df = 3, *p* = 0.097
1	40 (15.6)	14 (9.8)	
2	9 (3.5)	2 (1.4)	
3	3 (1.2)	0	
Adenomatous nodules, *n* (%)			
No	218 (84.8)	116 (81.1)	*χ2* = 0.92, df = 1, *p* = 0.399
Yes	39 (15.2)	27 (18.9)	
Colloidal nodules, *n* (%)			
No	81 (31.5)	49 (34.3)	*χ2* = 0.32, df = 1, *p* = 0.579
Yes	176 (68.5)	94 (65.7)	
Papillary carcinoma, *n* (%)			
No	203 (79.0)	142 (99.3)	*χ2* = 0.46, df = 1, *p* = 0.518
Yes	54 (21.0)	26 (18.2)	
Follicular carcinoma, *n* (%)			
No	257 (100)	399 (99.3)	*χ2* = 1.80, df = 1, *p* = 0.358
Yes	0	1 (0.7)	
Medullary carcinoma, *n* (%)			
No	254 (98.8)	143 (100)	*χ2* = 1.68, df = 1, *p* = 0.556
Yes	3 (1.2)	0	
Anaplastic carcinoma, *n* (%)			
No	257 (100)	143 (100)	
Autoimmune thyroiditis, *n* (%)			
No	234 (91.1)	129 (90.2)	*χ2* = 0.08, df = 1, *p* = 0.857
Yes	23 (8.9)	14 (9.8)	
Calcium day one, mmol/L, mean (SD)	2.05 (0.12)	2.24 (0.11)	< 0.001
MD (IQR)	2.05 (0.16)	2.25 (0.13)	
Ionized calcium day one, mmol/L, mean (SD)	0.96 (0.07)	1.06 (0.07)	< 0.001
MD (IQR)	0.96 (0.08)	1.05 (0.09)	
PTH, ng/L (pg/mL), mean (SD)	24.42 (18.27)	39.97 (17.50)	< 0.001
MD (IQR)	21.47 (29.24)	38.09 (20.86)	
Calcium day two, mmol/L, mean (SD)	1.96 (0.12)	2.20 (0.08)	< 0.001
MD (IQR)	1.99 (0.18)	2.19 (0.11)	
Ionized calcium day two, mmol/L, mean (SD)	0.94 (0.07)	1.06 (0.07)	< 0.001
MD (IQR)	0.94 (0.09)	1.05 (0.08)	
Revision for bleeding, *n* (%)			
No	254 (98.8)	142 (99.3)	*χ2* = 0.20, df = 1, *p* = 1.000
Yes	3 (1.2)	1 (0.7)	
Wound infection, *n* (%)			
No	257 (100)	143 (100)	
Wound hematoma, *n* (%)			
No	249 (96.9)	140 (97.9)	*χ2* = 0.35, df = 1, *p* = 0.753
Yes	8 (3.1)	3 (2.1)	
Bleeding during surgery, *n* (%)			
No	256 (99.6)	143 (100)	*χ2* = 0.56, df = 1, *p* = 1.000
Yes	1 (0.4)	0	
Clinical voice hoarseness, *n* (%)			
No	245 (95.3)	139 (97.2)	*χ2* = 0.84, df = 1, *p* = 0.434
Yes	12 (4.7)	4 (2.8)	
Recurrent laryngeal nerve paralysis on one side, ORL, *n* (%)			
No	252 (98.1)	140 (97.9)	*χ2* = 0.84, df = 1, *p* = 0.434
Yes	5 (1.9)	3 (2.1)	
Recurrent laryngeal nerve paralysis on both sides, ORL, *n* (%)			
No	257 (100)	143 (100)	
Clinical manifestation of hypocalcemia, *n* (%)			
None	197 (76.7)	143 (100)	*χ2* = 39.27, df = 2, *p* < 0.001
Day 1	14 (3.5)	0	
Day 2	46 (17.9)	0	
Hypocalcemia without clinical manifestation, *n* (%)			
No	60 (23.3)	143 (100)	*χ2* = 215.99, df = 1, *p* < 0.001
Yes	197 (76.7)	0	
Hypocalcemia - Chvostekʼs symptoms, *n* (%)			
No	243 (94.6)	143 (100)	*χ2* = 8.07, df = 1, *p* = 0.004
Yes	14 (5.4)	0	
Hypocalcemia - Trousseau’s symptoms, *n* (%)			
No	249 (96.9)	143 (100)	*χ2* = 4.54, df = 1, *p* = 0.033
Yes	8 (2.0)	0	
Hypocalcemia - Numbness of fingers, *n* (%)			
No	199 (77.4)	143 (100)	*χ2* = 37.75, df = 1, *p* < 0.001
Yes	58 (22.6)	0	
Treatment commenced in hospital with calcium and vitamin D preparations after surgery, *n* (%)			
None	23 (8.9)	139 (97.2)	*χ2* = 297.04, df = 4, *p* < 0.001
Day 1	15 (5.8)	0)	
Day 2	33 (12.8)	0	
Day 3	5 (1.9)	0	
On discharge	181 (70.3)	4 (2.8)	

^a^Continuous variables are presented as means and standard deviations (*SD*) & medians and interquartile ranges (*IQR*); categorical variables are presented as *n* (%). ^b^Student t test; ^c^Mann-Whitney U test. ^*^ *p* < 0.05, z test, ^**^ *p* < 0.01, ^***^ *p* < 0.001. *PTH* Parathyroid hormone, *ORL* otorhinolaryngologist

Comparison of the patients with diagnosed hypocalcemia following surgery and the patients without diagnosis of hypocalcemia showed that the following variables were significantly related to the development of hypocalcemia: decreased pre-operative Ca (*p* < 0.001), and decreased pre-operative iCa (*p* < 0.001). Our study did not showed an increased risk of postoperative hypocalcemia in the case of malignancy: papillary carcinoma (*p* = 0.518), follicular carcinoma (*p* = 0.358), medullary carcinoma (*p* = 0.556), and bilateral central neck dissection (*p* = 1.000), wound hematoma (*p* = 0.753), bleeding during surgery (*p* = 1.000), revision for bleeding (*p* = 1.000). These data are presented in Table 2.

Univariate analysis revealed that thyrotoxicosis <10 years before surgery was a predisposing factor for hypocalcemia (OR 1.65, 95% CI 1.01–2.70, *p* = 0.046). The number of PGs observed during surgery was also statistically associated with post-operative hypocalcemia (OR 0.52, 95% CI 0.38–0.70, *p* < 0.001). Ligation of the trunk of the left inferior thyroid artery (OR 2.04, 95% CI 1.27–3.29, *p* = 0.003) and the trunk of the right inferior thyroid artery (OR 2.37, 95% CI 1.47–3.82, *p* < 0.001) were statistically significant factors related to post-operative hypocalcemia. The number of PGs autotransplanted during surgery was also significantly associated with post-TT hypocalcemia (OR 1.87, 95% CI 1.12–2.97, *p* = 0.015). Finally, PTH on day one following the surgery was a statistically significant predictor of post-operative hypocalcemia (OR 0.96, 95% CI 0.94–0.97, *p* < 0.001). This data is presented in Table 3.

**Table 3:** Associations between patient characteristics and risk of hypocalcemia - Univariate model

Variable	OR	95% CI	*p*
Thyrotoxicosis before surgery: no	1		0.132
<10 years	1.65	1.01–2.70	0.046
>10 years	0.99	0.37–2.63	0.982
Thyroidectomy without lymphadenectomy	0.63	0.35–1.13	0.118
Thyroidectomy with one sided central lymphadenectomy	1.62	0.86–3.04	0.134
Thyroidectomy with central lymphadenectomy on both sides	1.31	0.33–5.13	0.702
Upper branch of left inferior thyroid artery, ligation	0.74	0.35–1.55	0.424
Lower branch of left inferior thyroid artery, ligation	0.62	0.28–1.38	0.241
Trunk of left inferior thyroid artery, ligation	2.04	1.27–3.29	0.003
Upper branch of right inferior thyroid artery, ligation	1.25	0.65–2.41	0.500
Lower branch of right inferior thyroid artery, ligation	1.90	0.84–4.31	0.125
Trunk of right inferior thyroid artery, ligation	2.37	1.47–3.82	<0.001
Number of darkened parathyroid glands at the end of surgery	0.98	0.69–1.39	0.925
Parathyroid gland hematoma	0.90	0.36–2.22	0.818
Adenomatous nodules	0.77	0.45–1.32	0.339
Colloidal nodules	1.13	0.73–1.75	0.574
Papillary carcinoma	1.20	0.71–2.01	0.498
Autoimmune thyroiditis	0.91	0.45–1.82	0.781
Graves’ disease	0.55	0.20–1.52	0.247
Calcium before surgery	0.01	1.01–0.57	<0.001
Calcium, day 1 after surgery	0.00	0.00–0.00	<0.001
Calcium, day 2 after surgery	0.00	0.00–0.00	<0.001
Ionized calcium before surgery	0.001	0.000–0.013	<0.001
Ionized calcium, day 1 after surgery	0.00	0.00–0.00	<0.001
Ionized calcium, day 2 after surgery	0.00	0.00–0.00	<0.001
PTH before surgery	1.00	0.99–1.01	0.854
PTH, day 1 after surgery	0.96	0.94–0.97	<0.001
25-hydroxyvitamin D before surgery	0.99	0.99–1.00	0.201
TSH before surgery	1.13	0.94–1.36	0.197
FT4 before surgery	0.98	0.91–1.06	0.625
FT3 before surgery	0.99	0.91–1.08	0.854

*PTH* Parathyroid hormone, *TSH* thyroid stimulating hormone, *FT3* free triiodothyronine, *FT4* free thyroxine

Unlike the univariate analysis, multivariate analysis revealed that older age (OR 1.05, 95% CI 1.01–1.09, *p* = 0.029) and female gender (OR 5.94, 95% CI 1.13–31.26, *p* = 0.035) were statistically significant independent predictors of post-operative hypocalcemia. See Table 4.

**Table 4:** Associations between patient characteristics and risk of hypocalcemia - Multivariate model

Variable	OR	95% CI	*p*
Age	1.05	1.01–1.09	0.029
Gender	5.94	1.13–31.26	0.035
Calcium, second day after surgery	0.00	0.00–0.00	<0.001

*OR* Odds ratio, *CI* confidence interval

## Discussion

Post-TT hypocalcemia is one of the most common complications [10]. According to the literature, post-TT hypocalcemia occurs in 50–68% of patients [3, 4]. In our study, post-TT hypocalcemia developed in 64.2% of the patients. In the literature, the majority of patients exhibit asymptomatic hypocalcemia and need no treatment, or only oral administration of Ca and vitamin D preparations [11]. In our study, 197 patients had asymptomatic hypocalcemia (49.3% of all the patients who participated in the study, or 76.7% of the patients with post-operative hypocalcemia). Less than half of the patients received no treatment after TT (161; 40.3%). The patients who received treatments were prescribed oral or intravenous preparations of Ca and vitamin D, depending on the time of expression and intensity of clinical symptoms (Table 2). Overall, 60 patients (15% of all the patients who participated in the study, or 23.3% of the patients with post-operative hypocalcemia) had one, two, or three clinical symptoms of hypocalcemia. In a multicenter study with 14,934 patients, Rosato et al. found that 10% of patients had symptomatic hypocalcemia [3]. However, that study included patients who had TT (64.3%) and smaller-scale surgeries on the thyroid (35.7%) [3]. In our study, decreased pre-operative Ca and iCa were statistically significant predictors of post-operative hypocalcemia (*p* < 0.001). Several studies have found that temporary post-operative hypocalcemia develops more often in patients who have a markedly decreased level of Ca before surgery [12–18]. However, a meta-analysis of six studies with 2493 patients did not reveal a statistically reliable association between pre-operative Ca and the frequency of temporary hypocalcemia [17–21]. A sharp decrease in Ca after TT is associated with temporary hypocalcemia [12, 22–24]. In a multicenter study with 1157 patients, Hallgrimsson et al. found that patients who experienced a 2–3% decrease in post-operative Ca in the 24 h following surgery, in comparison with the pre-operative level of Ca, had a 94% chance of developing temporary hypocalcemia [25]. Changes in the levels of Ca in the blood during the first 24 h following surgery allow prediction of temporary hypocalcemia with 19–91% sensitivity [26–30]. A blood Ca concentration of 1.88 mmol/L or less during the first 24 h after surgery has been associated with permanent hypocalcemia [31]. Two other studies have demonstrated that there is an increased risk of developing permanent hypocalcemia if the level of Ca in the blood remains at 2 mmol/L or less, 1–3 weeks after surgery [32, 33]. Tartaglia et al. revealed that measurement of iCa was more reliable than measurement of Ca in post-TT patients, in the immediate and long-term follow-up [34].

Based on our data, decreased PTH during the first 24 h after surgery was a statistically reliable predictor of post-operative hypocalcemia (*p* < 0.001). In many other studies, a decrease in blood PTH following TT was found to result in possible development of temporary hypocalcemia [12, 35–39]. The systematic overview and meta-analysis conducted by Edafe et al. showed that patients with a decrease in post-operative PTH, to a level of 6–35 pg/mL, 1 h to 1 day following surgery, had a 69–100% chance of developing temporary hypocalcemia [9].

According to our data, the number of PGs identified during the surgery was a statistically significant predictor of the development of hypocalcemia. The greater the number of PGs found during surgery, the lower the chance of hypocalcemia. The study by Thomusch et al. determined that permanent post-operative hypocalcemia was more likely to develop if less than two PGs were found during surgery [40]. On the other hand, other studies have found that more PGs found during surgery may be associated with temporary hypocalcemia [12, 25, 41].

In our study, the number of PGs autotransplanted during surgery was a statistically reliable predictor of the development of post-TT hypocalcemia (*p* = 0.015). The greater the number of autotransplanted PGs, the higher the chance of hypocalcemia. Several studies have shown that autotransplantation of one or more PGs results in a far higher chance of hypocalcemia [12, 17, 18, 42]. However, several other studies showed no association between autotransplantation of PGs and permanent hypocalcemia [18, 25, 31, 43].

In our study, thyrotoxicosis <10 years before surgery was found to be a statistically reliable predictor of post-TT hypocalcemia (*p* = 0.046). Thyrotoxicosis >10 years before surgery was not significantly associated with post-TT hypocalcemia (*p* = 0.982); however, there were only 18 such patients (4.5%) in our study. Based on our data, Graves’ disease was not a statistically significant predictor of post-TT hypocalcemia (*p* = 0.247), but again, it was only diagnosed in 21 patients (5.3%). The multivariate analysis by Thomusch et al. demonstrated that, in patients with Graves’ disease, both temporary and permanent hypocalcemia developed more often following TT [40]. Further, a meta-analysis of four studies with 6681 patients showed a greater frequency of post-operative temporary hypocalcemia in patients with Graves’ disease [40, 44–46].

Ligation of the trunk of the left inferior thyroid artery (*p* = 0.003) and the trunk of the right inferior thyroid artery (*p* < 0.001) were found to be statistically reliable predictors of hypocalcemia in our study. In a study of 1411 patients, Park et al. found that saving vessels supplying blood to the thyroid gland during surgery reduced the frequency of both temporary and permanent hypocalcemia in univariate (*p* = 0.01) and multivariate (*p* = 0.02) analyses [47]. According to Lee et al., saving veins on both sides of the thyroid may reduce post-TT hypocalcemia [48]. It has long been accepted that post-TT hypocalcemia depends on the experience of the surgeon and the surgical technique used [49, 50]. Our study identified some risk factors related to the surgical technique; hence, it is important not only to identify important factors following surgery that may influence development of hypocalcemia, but also to reconsider the surgical technique in order to minimize this risk, if possible.

In our study, the frequency analysis showed that neither gender (*p* = 0.351) nor age (*p* = 0.207) had a significant impact on development of hypocalcemia. However, in the multivariate analysis, female gender (*p* = 0.035) and older age (*p* = 0.029) were statistically significant predictors. There is no definitive evidence on the impact of age on the development of post-operative hypocalcemia in the literature. Some studies suggest that temporary post-operative hypocalcemia is more common in younger patients [15, 25], while others suggest it is more common in older patients [19, 21]. A meta-analysis of five studies with 2576 patients revealed no significant association between patient age and temporary hypocalcemia [15, 17–19, 21]. While a meta-analysis of 10 studies involving 3443 patients showed that temporary post-operative hypocalcemia was more common in women [12, 15, 17–21, 23, 41, 51].

## Conclusions

This study demonstrates that there is a number of different patient (gender, age, and thyrotoxicosis <10 years before surgery) and surgical (number of PGs found observed surgery, decreased Ca and iCa before surgery, PTH on day one following surgery, and ligation of the trunk of the left and right inferior thyroid artery) risk factors predictive of the development of hypocalcemia following TT. Our study suggests that it is important not only to identify factors following surgery that may predict development of hypocalcemia, but also to reconsider the surgical technique, in order to minimize this risk, if possible. Identification of known risk factors post-operatively could allow for early detection and effective treatment of these patients.

## Abbreviations

25-hydroxy Vit D: 25-hydroxyvitamin D
Ca: Calcium
CIs: Confidence Intervals
iCa: ionized Calcium
LR: Likelihood Ratio
ORs: Odds Ratios
PGs: Parathyroid Glands
PTH: Parathyroid Hormone
TT: Total Thyroidectomy
